# Effect of Oral Pre-Meal Administration of Betaglucans on Glycaemic Control and Variability in Subjects with Type 1 Diabetes

**DOI:** 10.3390/nu9091004

**Published:** 2017-09-12

**Authors:** Anders Frid, Andrea Tura, Giovanni Pacini, Martin Ridderstråle

**Affiliations:** 1 Department of Endocrinology, Skåne University Hospital, 205 04 Malmö, Sweden; Anders.Frid@skane.se; 2 Metabolic Unit, CNR Institute of Neuroscience, 35127 Padova, Italy; andrea.tura@cnr.it (A.T.); giovanni.pacini@cnr.it (G.P.); 3 Steno Diabetes Center, 2820 Gentofte, Denmark; 4 Department of Clinical Sciences, Lund University, 205 04 Malmö, Sweden

**Keywords:** betaglucan, viscous fiber, diabetes, glucose control, glucose variability

## Abstract

We conducted a double-blind placebo-controlled crossover pilot study to investigate the effect of oat betaglucans (β-glucan) on glycaemic control and variability in adults with type 1 diabetes (T1D; *n* = 14). Stomacol^®^ tablets (1.53 g of β-glucan) or placebo (Plac) were administered three times daily before meals for two weeks. Glucose levels were monitored during the second week by continuous glucose monitoring (CGM). There was an increase in basic measures of glycaemic control (maximal glucose value 341 ± 15 vs. 378 ± 13 mg/dL for Plac and β-glucan, *p* = 0.004), and average daily risk range (62 ± 5 vs. 79 ± 4 mg/dL for Plac and β-glucan, *p* = 0.003) favouring Plac over β-glucan, but no increase in the M-value (the weighted average of the glucose values) or other more complex measures. Basic measures of glucose variability were also slightly increased during β-glucan treatment, with no difference in more complex measures. However, glycaemic variability increased between the first and last two CGM days on Plac, but remained unchanged on β-glucan. In conclusion, in this pilot study we were unable to demonstrate a general positive effect of β-glucan before meals on glucose control or variability in T1D.

## 1. Introduction

Betaglucans (β-glucan) are non-digestible fibres that form a viscous gel in the gastrointestinal tract [1]. They are approved by the US Food and Drug Administration for lowering low density lipoprotein-cholesterol at a dose of 3 g daily [1]. The mechanism for this effect has been suggested to involve sequestering of bile acids, analogous to regular bile acid sequestrates [2,3]. Meanwhile, the gelling ability due to the high viscosity of β-glucans has also been suggested to positively influence postprandial glucose, and although data have been inconclusive, this ability is also recognised by the European Food Safety Authority [4,5]. Oat β-glucan has a higher molecular weight than barley β-glucan [6]. Its high viscosity even at low concentrations (1%) is believed to delay gastric emptying, slow intestinal transit, and postpone glucose and sterol absorption, all of which may contribute to attenuated postprandial plasma glucose and insulin levels, as has been observed in subjects with type 2 diabetes [7].

Postprandial glucose excursions and glucose variability are substantial challenges to people with diabetes, particularly those on insulin treatment, in part due to variable gastric emptying and intestinal transit time that can be secondary to either variable glucose levels themselves or to diabetic neuropathy [8,9]. Here we aimed to test the hypothesis that oat β-glucan might positively affect post-meal glucose excursions due to their gelling ability in subjects with type 1 diabetes (T1D).

## 2. Materials and Methods 

### 2.1. Study Set-Up and Subjects

**Figure 1:**
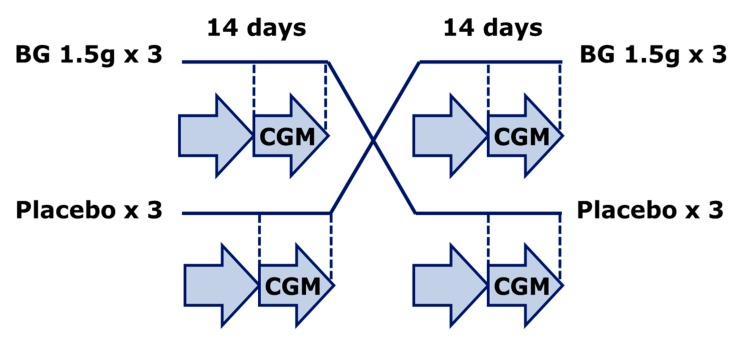
Study setup. A double blind placebo controlled crossover design with patients with type 1 diabetes (T1D) randomized to either betaglucan (BG) tablets (1.5 g before meals three times daily) or placebo for 14 days followed by a one-week wash-out period and a new 14-day treatment period. Plasma glucose variability was assessed by blinded continuous glucose monitoring (CGM) during the last 7 days of each treatment period.

We conducted a double blind placebo-controlled crossover study in 14 adult patients with T1D at the Department of Endocrinology, Skåne University Hospital (Malmö, Sweden), as shown in Figure 1. In this pilot study both patients with and without previous problems with variable glucose excursions were informed and invited to participate during routine out-patient visits to the clinic. A diagnosis of T1D, insulin treatment, and informed consent were the only inclusion criteria. However, none of the study participants were taking any kind of medication that would influence gastric emptying or intestinal transit, including glucagon-like peptide-1 agonists or sodium-glucose co-transporter-2 antagonists. One female subject was pre-menopausal. It is unknown weather this could influence glucose variability but there was no difference in her glucose readings between the two periods.

The study was designed to be as pragmatic as possible with respect to both timing of dose and the dose itself where tolerability/abdominal discomfort is the most common limitation. One Stomacol^®^ (Brainbridge Commerce AB, Gothenburg, Sweden) tablet (1.53 g β-glucan) or placebo (Plac) was administered three times daily before main meals for two weeks. Patients were instructed to chew on the tablets (2.9 g total weight, 7.8 × 20 mm diameter) until dissolved, and to then swallow them with a glass of water prior to having breakfast, lunch, and dinner. There was no formal control of compliance but patients were asked whether they took the tablets regularly and there was no report of missing doses unless patients ended the study prematurely (one patient). β-glucan and Plac were kind gifts from Brainbridge Commerce AB (Gothenburg, Sweden). The first two-week period was followed by an at least two-week long washout period before the second two-week period on crossover treatment. The patients were asked not to change their daily life habits or eating, but to measure plasma glucose and adjust insulin doses as appropriate just as they normally would. Primary endpoints were glycaemic control and variability. Glucose levels were monitored during the second week by continuous glucose monitoring (CGM; Medtronic iPro2™ with the Enlite^®^ (Medtronic, Minneapolis, MN, USA) sensor; a kind gift from Medtronic AB, Solna, Sweden). Both patients and health care providers were blinded to these readings and patients were instructed to carry on living as previously (including reporting hypoglycemia and adjusting insulin dose according to routine finger prick glucose measures). According to the rules of the ethical committees posted by the local committee at Lund University (Lund, Sweden), all forms of treatment quality assurance are exempt from ethical committee approval. Since this research involved comparing blood glucose between groups with different intake of a substance normally found in the diet of a person with diabetes without introducing anything that could be considered outside of the normal diet, the study was considered to fulfil the criteria of quality assurance of blood glucose control rather than being a study of the medical effects of a new treatment.

### 2.2. Calculation of Indices for Continuous Glucose Monitoring (CGM) Data Analysis

The indices of glycaemic control [10,11,12,13] describe to what extent the glucose data tend to remain near a target value or in a target range. There are both basic indices of descriptive statistics, and more complex indices. As regards to the former, the calculated indices were glucose mean, maximum, minimum, 50th percentile (median) as well as other percentiles, and percentages of glucose values in a target range (4.4–11.1 mmol/L, i.e., 80–200 mg/dL), and below and above a target value (4.4 and 11.1 mmol/L, respectively). More complex indices were also measured. One was the glycemic risk assessment diabetes equation (GRADE), where glucose values were transformed to yield a continuous curvilinear response with a nadir of 5.5 mmol/L and high adverse weighting to hyperglycemia and hypoglycemia using the equation

GRADE = 425 × {log_10_[log_10_(*Gluc_n_*)] + 0.16}^2^ (1)

with *Gluc_n_* in mmol/L. Then, the average value was taken. Another measure was the M-VALUE, a weighted average of the glucose values with progressively larger penalties for more extreme values. The corresponding equation is

M-VALUE = |10 × log_10_ (*Gluc_n_/*IGV)|^3^ (2)

where IGV is the ideal glucose value, typically assumed, as in this study, as equal to 6.7 mmol/L (120 mg/dL). Again, an average value was then taken. A third measure was the hypoglycemia index, which is the weighted average of hypoglycemic values. If the blood glucose value is lower than a given threshold, the index is:

*Hypo_index* = (LLTR − *Gluc_n_*)^2.0^/30 (3)

with *Gluc_n_* and LLTR (Lower Limit of the Target Range) in mg/dL (typically, LLTR = 80 mg/dL). The hyperglycemia index, also used, is the weighted average of hyperglycemic values. If the blood glucose value is higher than a given threshold, the index is:

*Hyper_index* = (*Gluc_n_* − ULTR)^1.1^/30 (4)

with *Gluc_n_* and ULTR (Upper Limit of the Target Range) in mg/dL (typically, ULTR = 140 mg/dL). Other measures were the index of glycaemic control (IGC), which is the *Hypo_index* + *Hyper_index*, and the low blood glucose index (LBGI), which is a transformation that normalizes the blood glucose scale:

LBGI = 1.509 × [(log_e_(*Gluc_n_*))^1.084^ − 5.381] (5)

for blood glucose values less than 112.5 mg/dL. Then, a risk value is assigned to each blood glucose reading as follows:

Risk(LBGI) = 10 × LBGI^2^ (6)

and finally, the average value is taken. The high blood glucose index (HBGI) was also obtained. Similar to the LBGI, this is a transformation to normalize the blood glucose scale for blood glucose values higher than 112.5 mg/dL. The expression of HBGI is the same as for LBGI. Finally, the average daily risk range (ADRR) is *LBGI* + *HBGI*, calculated with the minimum and the maximum glucose value, respectively.

The indices of glycaemic variability [10,11,12,13] measure to what extent CGM data tend to oscillate. The higher the variability, the higher the value of such indices. Some basic indices that we calculated were the glucose standard deviation (SD), the coefficient of variation, and the total and the interquartile range. More complex indices included the J-INDEX, which is a combination of information from mean and SD of all glucose values, calculated as

J-INDEX = 0.001 × (mean + SD)^2^ (7)

and continuous overlapping net glycaemic action (CONGA_n_), which is the SD of the difference between values obtained exactly *n* minutes apart. Typically, *n* is equal to 60 min (or its multiples), but in this case we performed the analysis over the glucose data available, despite the fact that the time interval between consecutive values was typically lower than one hour. Also used was the lability index:

(8) $$LI=\overset{N}{\underset{n=1}{\sum }}\frac{{\left(Gluc_{n+1}-Gluc_{n}\right)}^{2}}{h_{n+1}-\;h_{n}}$$

where *Gluc_n_* (in mmol/L) is the *n*-th glucose value, and *h_n_* is the time when that value was collected (similarly to *Gluc_n+1_* and *h_n+1_*); *N* is the total number of readings. Typically, *h_n+1_* and *h_n_* are at least one hour apart, but we again exploited all the CGM data available. We also computed the mean, instead of the sum. Another index was the mean amplitude of glycaemic excursion (MAGE), which is the arithmetic mean of the glycaemic excursions that are greater than one SD. MAGE (pos) and MAGE (neg) consider the positive and the negative excursions, respectively. The shape index is based on the calculation of the point-by-point second-order derivative of the glucose curve; then, absolute value of each derivative is taken, and the average over all values is calculated. The autocorrelation index considers to what extent the glucose values tend to repeat or change during time, and the autocorrelation sequence is computed as

(9) $$\mathrm{Ad}(m)=\frac{1}{N-|m|}\sum_{i=1}^{N-|m|}x(i)\cdot x(i+m)$$

where *x* in this case is glucose, *N* is the total number of samples and *m* is the time lag expressed as number of samples; then the sequence is normalized to Ad(1), and average value is calculated to get the autocorrelation index.

### 2.3. Statistical Analysis

After testing for normality of distribution, values of the indices were logarithmically transformed, and possible differences between the groups were assessed by paired Wilcoxon test. *p* < 0.05 was considered statistically significant. Values are reported as mean ± standard deviation (SD) or standard error (SE), unless otherwise specified.

## 3. Results

One patient stopped the study prematurely and the glucose sensor reading of another failed, leaving 12 evaluated patients: 4 females and 8 males, aged 47 ± 12 years, with age at onset of T1D 25 ± 17 years and T1D duration 22 ± 16 years (mean ± SD). In general, β-glucan and Plac were well tolerated. Table 1 summarizes the CGM data from the last 7 days of the study periods on Plac and β-glucan, respectively. There were no general improvements in favour of β-glucan over Plac. Instead, there was a significant increase in some basic measures of glycaemic control such as maximal glucose and average daily risk range, but none in glycaemic risk assessment diabetes equation or M-value, which are more complex measures of glycaemic control as measured by CGM. Basic measures of glucose variability such as standard deviation were also slightly increased during β-glucan, with no difference in more complex measures (Table 1).

Since participation in a study might result in improved glycaemic control and lesser glycaemic variability by itself, for instance due to more frequent glucose measuring and careful insulin dosing, we were also interested to see any trends over time. Indeed, whereas glucose variability tended to increase between the first and last two CGM days on Plac (coefficient of variation from 34.3 ± 1.9 to 39.1 ± 3.0%; *p* < 0.05), it remained unchanged on β-glucan (from 38.2 ± 3.2 to 38.9 ± 2.1%; *p* = ns (non-significant)), suggesting a time dependent positive effect in maintaining a stable glucose level.

## 4. Discussion 

From this small-scale pilot study on the effect of pre-meal β-glucan administration on glycaemic control and glucose variability we conclude that the ingestion of 1.5 g β-glucanthree times daily for two weeks by patients with T1D was safe and well tolerated. However, with regards to the primary outcome of glycemic control and glucose variability there was clearly no benefit in favour of pre-meal β-glucan. The study dose, potentially being too low, dosing timing, potentially being too close to the meals, study duration, being too short, and lack of controlling for a range of factors that occur in real life might be important factors to consider for future interventions. With regard to the quality of the β-glucan preparation, it also seems important that a high viscosity gel be produced to affect post-prandial glycaemia [14].

Soluble dietary fibres have been shown to attenuate the postprandial rise in blood glucose levels and even reduce the risk of type 2 diabetes and cardiovascular disease [1,2,3,4,15,16]. This effect seems to be related to its rheological properties including viscosity, which in turn depends on processing methodology with enzymatic methods being more advantageous than aqueous methods [12]. The increase in viscosity of the stomach contents is thought to delay gastric emptying and reduce the mixing of food with digestive enzymes, which in turn would retard glucose absorption. An alternative explanation to an effect on glycaemia *per se* could be an effect on satiety and dietary intake [17,18]. Our study was not designed to investigate such alternatives.

While our findings were not as straightforward, a recent pilot in healthy volunteers suggests that pre-meal β-glucan ingestion might be a valid strategy to control postprandial glucose excursions [19]. A major difference between our studies is the lack of normal glucose regulation by endogenous insulin in the subjects participating here. In addition, rather than looking at a test-meal situation we investigated the potential effect on 24-h glycaemia by CGM. From our data, we conclude that for most patients with T1D, the effect of pre-meal β-glucan will not be as predictable as it would in a person with normal glucose tolerance and/or insulin and incretin secretion [20]. It is important to stress a major similarity in the studies being the instruction to ingest β-glucan prior to eating to allow sufficient time for gelling since β-glucan has otherwise been shown to be ineffective in this respect [21,22]. This concept of “pre-load” has also been extensively studied in type 2 diabetes [23,24,25]. 

There are several other important limitations to our study. It was small-scale and short-term, and we included a general population of patients with T1D. This means that there is limited power and that one should be careful with the generalizability of our findings. In fact, one particular observation points to that longer observation times may be advisable for future studies. Comparing the two first and two last of the seven days with CGM there was a significant increase in glucose variability on placebo, as opposed to no change on β-glucan. Given the fact that the placebo effect of participating in a study on glucose control where so many different factors contribute is expected to be fairly strong, this finding is not surprising and may point to a positive long-term effect of pre-meal β-glucan. Another limitation of the study is the choice of β-glucan dose. In fact, 1.5 g three times daily was chosen as a trade-off between expected tolerability on the one hand and the effect on cholesterol on the other, but it is quite possible that a higher dose, e.g. related to body mass or meal carbohydrate content but still limited by the expected gastrointestinal discomfort, may be more effective [1,5]. From a technical point of view there is also the choice of CGM to evaluate the effect of β-glucan. The length of the study did not allow evaluation of classical long-term parameters such as hemoglobin A1c but there are other emerging techniques one may consider [26].

## 5. Conclusions

In conclusion, pre-meal ingestion of 1.5 g β-glucan three times daily for a two-week period was well tolerated and safe in subjects with T1D but did not lead to general improvements in glycaemic control or glucose variability in this small-scale pilot study.

## Figures and Tables

**Table 1:** Glycaemic control and glucose variability measures during the last 7 days of a two-week period on either placebo or betaglucan tablets three times daily.

CGM Parameter	Placebo	Betaglucan	*p*-Value
Mean glucose (mg/dL)	175 ± 10	176 ± 9	0.6
Maximal glucose (mg/dL)	341 ± 15	378 ± 13	0.004
Minimal glucose (mg/dL)	59 ± 5	50 ± 4	0.07
80–200 mg/dL range (% of values)	59 ± 4	57 ± 4	0.4
<80 mg/dL (% of values)	8 ± 2	10 ± 2	0.5
>200 mg/dL (% of values)	33 ± 5	34 ± 4	0.7
ADDR (unitless)	62 ± 5	79 ± 4	0.003
GRADE (unitless)	11 ± 1.1	12 ± 1.0	0.5
M-value (unitless)	19 ± 2.9	21 ± 3.3	0.5
Standard deviation (mg/dL)	65 ± 5	72 ± 4	<0.05
Coefficient of variation (%)	38 ± 2	41 ± 1	0.2
Glucose Range (mg/dL)	283 ± 14	329 ± 12	0.003
J-index ([mg/dL] ^2^)	60 ± 6	63 ± 6	0.3
CONGA (mg/dL)	5.3 ± 0.6	5.2 ± 0.4	0.9
MAGE (mg/dL)	120 ± 9	119 ± 10	0.9
Lability Index ((mg^2^)/h)	0.82 ± 0.09	0.87 ± 10	0.8
Shape Index (10^−3^ (mg/dL)/min^2^)	3.20 ± 0.15	3.06 ± 0.13	0.4
Autocorrelation (unitless)	0.22 ± 0.02	0.28 ± 0.03	0.1

Data are given as mean ± standard error. Wilcoxon test for differences were used throughout. CGM: continuous glucose measuring, ADDR: average daily risk range, GRADE: glycaemic risk assessment diabetes equation, CONGA: continuous overlapping net glycaemic action, MAGE: mean amplitude of glycaemic excursion. See Methods for further explanations of indices.

## References

[1] Jenkins D.J., Kendall C.W., Vuksan V., Vidgen E., Parker T., Faulkner D., Mehling C.C., Garsetti M., Testolin G., Cunnane S.C. (2002). Soluble fiber intake at a dose approved by the US Food and Drug Administration for a claim of health benefits: Serum lipid risk factors for cardiovascular disease assessed in a randomized controlled crossover trial. Am. J. Clin. Nutr..

[2] Othman R.A., Moghadasian M.H., Jones P.J. (2011). Cholesterol-lowering effects of oat β-glucan. Nutr. Rev..

[3] Gunness P., Michiels J., Vanhaecke L., de Smet S., Kravchuk O., van de Meene A., Gidley M.J. (2016). Reduction in circulating bile acid and restricted diffusion across the intestinal epithelium are associated with a decrease in blood cholesterol in the presence of oat β-glucan. FASEB J..

[4] Shen X.L., Zhao T., Zhou Y., Shi X., Zou Y., Zhao G. (2016). Effect of Oat β-Glucan Intake on Glycaemic Control and Insulin Sensitivity of Diabetic Patients: A Meta-Analysis of Randomized Controlled Trials. Nutrients.

[5] Panel on Dietetic Products, Nutrition and Allergies (NDA) (2011). Scientific Opinion on the substantiation of health claims related to beta-glucans from oats and barley and maintenance of normal blood LDL-cholesterol concentrations (ID 1236, 1299), increase in satiety leading to a reduction in energy intake (ID 851, 852), reduction of post-prandial glycaemic responses (ID 821, 824), and “digestive function” (ID 850) pursuant to Article 13(1) of Regulation (EC) No 1924/2006. EFSA J..

[6] Cloetens L., Ulmius M., Johansson-Persson A., Åkesson B., Önning G. (2012). Role of dietary beta-glucans in the prevention of the metabolic syndrome. Nutr. Rev..

[7] Butt M.S., Tahir-Nadeem M., Khan M.K.I., Shabir R., Butt M.S. (2008). Oat: Unique among cereals. Eur. J. Nutr..

[8] Škrha J., Šoupal J., Škrha J., Prázný M. (2016). Glucose variability, HbA1c and microvascular complications. Rev. Endocr. Metab. Disord..

[9] Frandsen C.S., Dejgaard T.F., Madsbad S. (2016). Non-insulin drugs to treat hyperglycaemia in type 1 diabetes mellitus. Lancet Diabetes Endocrinol..

[10] Rodbard D. (2009). Interpretation of continuous glucose monitoring data: Glycemic variability and quality of glycemic control. Diabetes Technol. Ther..

[11] Rodbard D. (2009). New and improved methods to characterize glycemic variability using continuous glucose monitoring. Diabetes Technol. Ther..

[12] Werzowa J., Pacini G., Hecking M., Fidler C., Haidinger M., Brath H., Thomas A., Säemann M.D., Tura A. (2015). Comparison of glycemic control and variability in patients with type 2 and posttransplantation diabetes mellitus. J. Diabetes Complicat..

[13] Tura A., Farngren J., Schweizer A., Foley J.E., Pacini G., Ahrén B. (2015). Four points pre-prandial self-monitoring of blood glucose for the assessment of glycemic control and variability in patients with type 2 diabetes treated with insulin and vildagliptin. Int. J. Endocrinol..

[14] Panahi S., Ezatagha A., Temelli F., Vasanthan T., Vuksan V. (2007). Beta-glucan from two sources of oat concentrates affect postprandial glycemia in relation to the level of viscosity. J. Am. Coll. Nutr..

[15] Ho H.V., Sievenpiper J.L., Zurbau A., Blanco Mejia S., Jovanovski E., Au-Yeung F., Jenkins A.L., Vuksan V. (2016). The effect of oat β-glucan on LDL-cholesterol, non-HDL-cholesterol and apoB for CVD risk reduction: A systematic review and meta-analysis of randomised-controlled trials. Br. J. Nutr..

[16] Tosh S.M. (2013). Review of human studies investigating the post-prandial blood-glucose lowering ability of oat and barley food products. Eur. J. Clin. Nutr..

[17] Rebello C.J., O’Neil C.E., Greenway F.L. (2016). Dietary fiber and satiety: The effects of oats on satiety. Nutr. Rev..

[18] Wanders A.J., van den Borne J.J.G.C., de Graaf C., Hulshof T., Jonathan M.C., Kristense M., Mars M., Schols H.A., Feskens E.J.M. (2011). Effects of dietary fibre on subjective appetite, energy intake and body weight: A systematic review of randomized controlled trials. Obes. Rev..

[19] Steinert R.E., Raederstorff D., Wolever T.M. (2016). Effect of Consuming Oat Bran Mixed in Water before a Meal on Glycemic Responses in Healthy Humans-A Pilot Study. Nutrients.

[20] Marathe C.S., Rayner C.K., Jones K.L., Horowitz M. (2013). Relationships between gastric emptying, postprandial glycemia, and incretin hormones. Diabetes Care.

[21] Jenkins D.J., Nineham R., Craddock C., Craig-McFeely P., Donaldson K., Leigh T., Snook J. (1979). Fibre in diabetes. Lancet.

[22] Fuessl S., Adrian T.E., Bacarese-Hamilton A.J., Bloom S.R. (1986). Guar in NIDD: Effect of different modes of administration on plasma glucose and insulin responses to a starch meal. Pract. Diabetes Int..

[23] Gentilcore D., Chaikomin R., Jones K.L., Russo A., Feinle-Bisset C., Wishart J.M., Rayner C.K., Horowitz M. (2006). Effects of fat on gastric emptying of and the glycemic, insulin, and incretin responses to a carbohydrate meal in type 2 diabetes. J. Clin. Endocrinol. Metab..

[24] Ma J., Stevens J.E., Cukier K., Maddox A.F., Wishart J.M., Jones K.L., Clifton P.M., Horowitz M., Rayner C.K. (2009). Effects of a protein preload on gastric emptying, glycemia, and gut hormones after a carbohydrate meal in diet-controlled type 2 diabetes. Diabetes Care.

[25] Ma J., Jesudason D.R., Stevens J.E., Keogh J.B., Jones K.L., Clifton P.M., Horowitz M., Rayner C.K. (2015). Sustained effects of a protein “preload” on glycaemia and gastric emptying over 4 weeks in patients with type 2 diabetes: A randomized clinical trial. Diabetes Res. Clin. Pract..

[26] Pandey R., Dingari N.C., Spegazzini N., Dasari R.R., Horowitz G.L., Barman I. (2015). Emerging trends in optical sensing of glycemic markers for diabetes monitoring. TrAC Trends Anal. Chem..

